# ILOOP – a web application for two-channel microarray interwoven loop design

**DOI:** 10.1186/1471-2164-9-S2-S11

**Published:** 2008-09-16

**Authors:** Mehdi Pirooznia, Ping Gong, Jack Y Yang, Mary Qu Yang, Edward J Perkins, Youping Deng

**Affiliations:** 1 Department of Biological Sciences, University of Southern Mississippi, Hattiesburg, MS, 39406, USA; 2 SpecPro Inc., Vicksburg, MS, 39180, USA; 3 Harvard University, Harvard Medical School, Cambridge, MA 02140-0888, USA; 4 National Human Genome Research Institute, National Institutes of Health, Bethesda, MD 20852, USA; 5 Environmental Laboratory, U.S. Army Engineer Research and Development Center, Vicksburg, MS, 39180, USA

## Abstract

Microarray technology is widely applied to address complex scientific questions. However, there remain fundamental issues on how to design experiments to ensure that the resulting data enables robust statistical analysis. Interwoven loop design has several advantages over other designs. However it suffers in the complexity of design. We have implemented an online web application which allows users to find optimal loop designs for two-color microarray experiments. Given a number of conditions (such as treatments or time points) and replicates, the application will find the best possible design of the experiment and output experimental parameters. It is freely available from .

## Background

Microarray technology is now widely used to address complex scientific questions and for studies of gene interactions. However, it is associated with a number of technical challenges. The high cost of microarrays plus the complex logistical issues associated with microarray studies, often require that compromises must be made in the number of samples analyzed. Replication of data is a fundamental and widely appreciated principle of design that is often sacrificed. Microarray users now acknowledge that "replication" means different things in the microarray context [1-3]. "Replication" might refer to (A) Spotting genes multiple times per array; (B) Hybridizing multiple arrays to the same RNA samples; and (C) Using multiple individuals of a certain variety or type. Replication types (A) and (B) are sometimes referred to as technical replication while type (C) represents biological replication in the classical statistical sense. Biological replicates can assess biological variability, which is essential, for instance, to surmise that the mean expression of a gene differs in two populations [4]. Three layers can be considered in a design of a two-color microarray experiment. Experimental units are at the top layer of the experiment, two RNA samples obtained from each unit are in the middle layer, and the arrangement of array elements on the slides would be placed at the bottom layer of the experiment [5].

**Figure 1:**
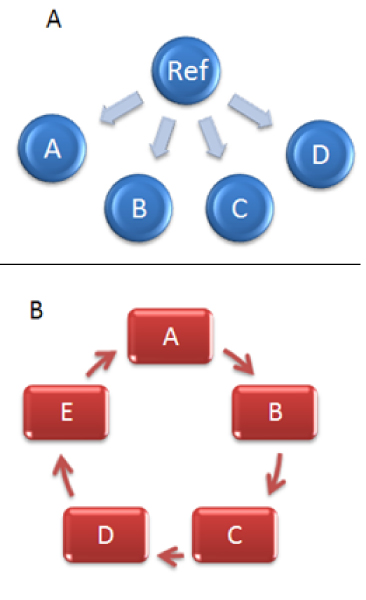
Combination of varieties with dyes for the reference (A) vs. loop design (B).

Certain decisions as to how many microarray slides will be used and which mRNA samples will be hybridized to each slide must be made in preparation of mRNA samples before carrying out a microarray experiment [6,7]. Kerr and Churchill [2] and Glonek and Solomon [8] suggested efficient designs for some common microarray experiments. The most commonly used design is the reference design (Figure 1-A). In this design, each condition of interest is compared with samples taken from a standard reference. This design allows an indirect comparison between the conditions, because the reference is common to all of the arrays. In contrast, a loop design (Figure 1-B) compares two conditions via a chain of other conditions or multiple-pairwise (interwoven loop) fashion [9,10].

The computation of variance in a loop design depends on the design and the number of samples. In the loop design, each sample is compared directly with other samples in a multiple-pairwise (circular) way. Most studies on microarray design suggest that the loop design of microarray experiments is more efficient than the reference design [11-13]. This approach has stronger statistical power than the reference design. Also, the entire dataset produced in a loop design is useful experimental information, while half the data produced in a reference design experiment is redundant.

In a cDNA microarray experiment, the foreground red and green intensities can be considered as *Rf *and *Gf *for each spot and the background intensities *Rb *and *Gb*. The background-corrected intensities will be *R *and *G *where *R *= *Rf*-*Rb *and *G *= *Gf*-*Gb*. M and A can be calculated as *M *= *log R*/*G and A *= 1/2 *log RG*. It is convenient to use base 2 logarithms for *M *and *A *so that *M *is units of 2-fold change. On this scale, *M *= 0 represents equal expression, *M *= 1 represents a 2-fold change between the RNA samples, *M *= 2 represents a 4-fold change, and so on. If treatment A is on array 1 and treatment B is on array 2, the contrast A-B is estimated by *M*_*i*1 _- *M*_*i*2 _with variance 4$\sigma_{i\varepsilon }^{2}$. With k replicates, the estimated contrast would have variance 4$\sigma_{i\varepsilon }^{2}$/k [14-17].

In loop design, using the optimal weighting, the variance of the contrast between adjacent treatments is $\sigma_{i\varepsilon }^{2}+\sigma_{ia}^{2}\sigma_{i\varepsilon }^{2}/2(\sigma_{ia}^{2}+\sigma_{i\varepsilon }^{2})$ while the variance of the contrast between diagonally opposite treatments is $\sigma_{i\varepsilon }^{2}+\sigma_{ia}^{2}\sigma_{i\varepsilon }^{2}/(\sigma_{ia}^{2}+\sigma_{i\varepsilon }^{2})$. Comparing these variances with variance of the contrast from a reference design with *K *replicates, $2\sigma_{i\varepsilon }^{2}/\text{k}+2\sigma_{ia}^{2}\sigma_{i\varepsilon }^{2}/\text{k}(\sigma_{ia}^{2}+\sigma_{i\varepsilon }^{2})$, it is clear that both of these variances in loop design are smaller than the variance of the contrast from a reference design with the same number of conditions and arrays, primarily because there are two replicates per sample, rather than one [17]. However one disadvantage of this method is that ratios observed across different pairwise comparisons are not immediately comparable and visualizations are more difficult [18,19].

**Figure 2:**
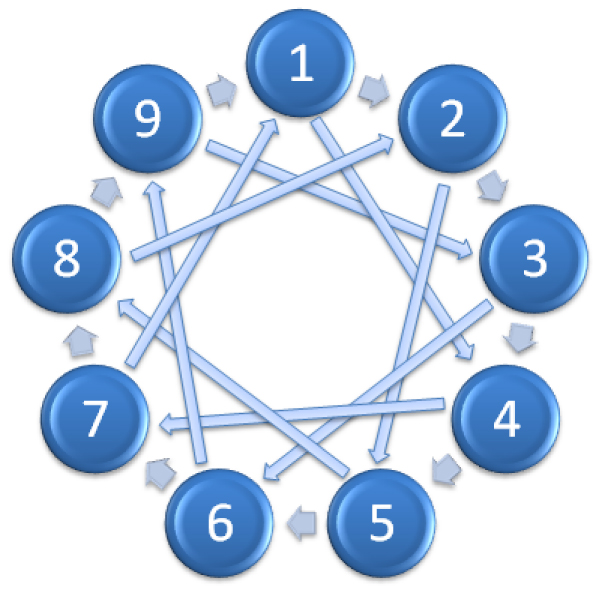
An example interwoven loop design with 18 arrays and 9 conditions.

Kerr and Churchill [2] noticed that a loop design stops being optimal when there are more than eight conditions. Therefore it has been suggested that the optimal design could be a form of an interwoven loop design. Figure 2 shows an example of interwoven loop design for an experiment with nine conditions (or time points) and 18 array slides [2,9,10].

Wit et al. [20] have developed an optimization algorithm that searches for the loop design which minimizes the A-optimality criterion. This is in fact an interwoven design. The interwoven design guarantees that each condition is measured equally often by either dye [21]. The Wit et al. optimization algorithm in fact allows one to input the number of conditions and the number of arrays one can afford to hybridize.

Currently biologists take a considerable amount of time to develop loop designs manually and the final design may not be optimized. To date there is no available tool for biologists to automatically design and visualize the interwoven loop for a microarray experiment. Development of such a tool will permit biologist to quickly generate different array hybridization loops, compare the cost and experiment design and efficiently design microarray studies so that robust statistical conclusion can be made.

## Implementation

Here we calculate the most optimal loop by considering the number of replicates and conditions. The main point is to generate an optimal number of arrays based on combination of conditions and replicates for two-dye microarray experiment.

**Figure 3:**
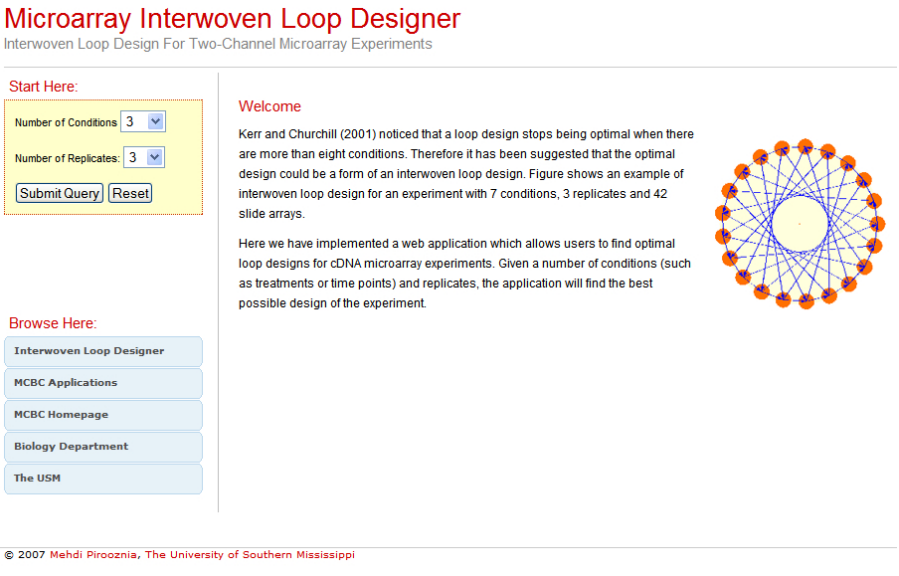
The web application screenshot.

The web application has been developed using PHP language on an open source Apache web server. It is freely available from  (Figure 3).

**Figure 4:**
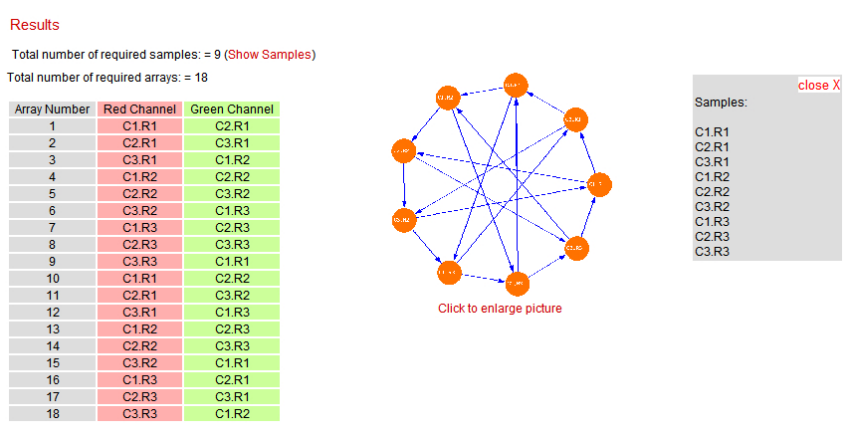
A screenshot of optimal interwoven loop table and graph.

**Figure 5:**
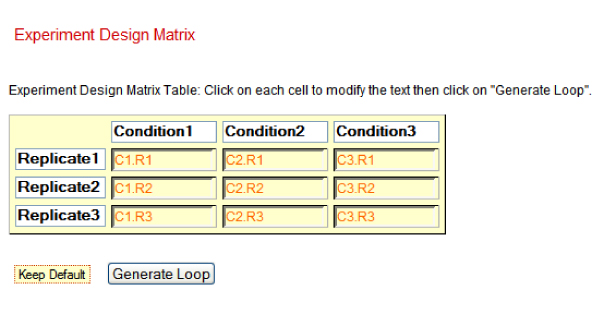
The experiment design matrix table.

Given a number of conditions (treatments/timepoints) and replicates, the program generates the optimal interwoven loop design. The start menu has two drop boxes, one for "Number of Conditions" and another for "Number of Replicates". By selecting the number of conditions and replicates, the application generates an experiment design matrix table (Figure 4 and 5). The following pseudo code represents the algorithm used for array construction from the experiment design matrix table:

$$\begin{array}{l} for\;(i=0;i<t-1;i++)\{\\ aray\;(sample[i],sample[i+1])\\ \}\\ array\;(sample[t-1],sample[0])\\ for\;(i=0;i<t;i++)\{\\ array\;(sample[i],sample[f\mathrm{mod}(i+1+c,t)])\\ \} \end{array}$$

Where *c *is the number of conditions and *t *is the total number of required sample (conditions × replicates).

In Figure 5, table cells represent a sample of the replicate with the corresponding condition. Here all cells are editable and user is able to change the condition and/or replicate's name. The application generates a visualization of optimal interwoven loop table and graph (Figure 4). The total number of required samples as well as the total number of required arrays is calculated. Any two spots connected with arrow in the graph in Figure 4 represent an array combined from red to green channel.

As an example figure 4 shows a screenshot of an optimal interwoven loop table and graph. In this case with 3 conditions and 4 biological replicates, the application calculates the total number of required samples, 12, and the total number of required arrays would be 24. A similar experiment with the reference design would require 24 arrays. However the loop design creates 4 technical replicates per sample and samples are always hybridized to different samples (biological replicates).

## Application

**Figure 6:**
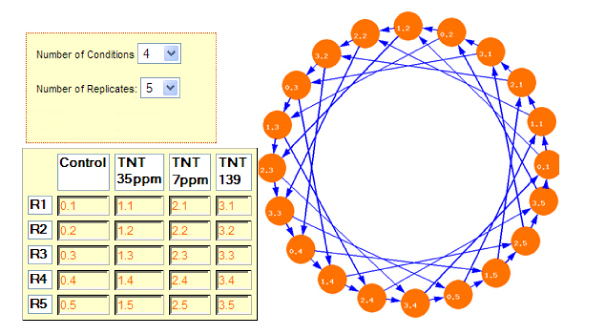
An interwoven loop hybridization schemes for 4 treatments with 5 independent biological replicates. Circles represent treatment samples. Sample code: 0.x = replicate × of solvent control worms; 1.x = replicate × of 10.6 mg TNT/kg soil treated worms; 2.x = replicate × of 2 mg TNT/kg soil treated worms; 3.x = replicate × of 38.7 mg TNT/kg soil treated worms; x = 1–5. Arrows represent array hybridizations between respective samples where the arrowhead indicates Alexa 647 dye labeling and the base of arrows indicate Cy3 dye labeling.

We evaluated the utility of the design application using a microarray designed to study the effect of chemical toxicity on earthworm [22]. Earthworms were exposed to three different concentration of TNT (2,4,6-trinitrotoluene). We used five biological replicates for each exposure. The application produced an optimal design of 40 arrays derived from 20 cDNA probes in accordance with an interwoven loop scheme as shown in Figure 6. cDNA samples from each biological replicate were labeled twice with a green channel fluorescence dye (Cy3) and twice with a red fluorescence channel dye (Alexa 647).

The hybridization experimental design tool proved simple to use and facilitated execution of the complex sample pairing required by this approach. A similar reference design would require 80 arrays to achieve 5 biological replicates over 4 conditions with 4 technical replicates compared to 40 required arrays in the optimal loop design in this experiment.

## Discussion

The main significance of this paper is introduction of a web application that implements loop design for microarray experiment. To date no online application has been available to achieve this goal. Such designs should be analyzed by treating the arrays as blocks of size 2 and analyzing the channels as individual observations.

Our web application will allow scientists to design and graph the optimal interwoven loop faster. They can quickly select the number of conditions and replicates and weight the number of samples and arrays in order to minimize the cost and complexity of the experiment as well as maximizing the efficiency of the experiment.

## Competing interests

The authors declare that they have no competing interests.

## Authors' contributions

MP and YD initiated the study. MP and YD designed and implemented the application, web server configuration and web application programming. PG and EJP provided the data. MP, YD, PG, and EJP drafted the original manuscript. YD, PG, JY, MY and EJP coordinated and directed the project. All authors have read and approved the final manuscript.
